# Computational approach to predict species-specific type III secretion system (T3SS) effectors using single and multiple genomes

**DOI:** 10.1186/s12864-016-3363-1

**Published:** 2016-12-19

**Authors:** Christopher K. Hobbs, Vanessa L. Porter, Maxwell L. S. Stow, Bupe A. Siame, Herbert H. Tsang, Ka Yin Leung

**Affiliations:** 1 Applied Research Laboratory, Faculty of Natural and Applied Sciences, Trinity Western University, 7600 Glover Road, Langley, BC Canada V2Y 1Y1; 2 Department of Biology, Faculty of Natural and Applied Sciences, Trinity Western University, 7600 Glover Road, Langley, BC Canada V2Y 1Y1; 3 State Key Laboratory of Bioreactor Engineering, East China University of Science and Technology, Shanghai, 200237 China

**Keywords:** Machine learning, Type III secretion system, Effector prediction, Gram-negative bacteria

## Abstract

**Background:**

Many gram-negative bacteria use type III secretion systems (T3SSs) to translocate effector proteins into host cells. T3SS effectors can give some bacteria a competitive edge over others within the same environment and can help bacteria to invade the host cells and allow them to multiply rapidly within the host. Therefore, developing efficient methods to identify effectors scattered in bacterial genomes can lead to a better understanding of host-pathogen interactions and ultimately to important medical and biotechnological applications.

**Results:**

We used 21 genomic and proteomic attributes to create a precise and reliable T3SS effector prediction method called Genome Search for Effectors Tool (GenSET). Five machine learning algorithms were trained on effectors selected from different organisms and a trained (voting) algorithm was then applied to identify other effectors present in the genome testing sets from the same (GenSET Phase 1) or different (GenSET Phase 2) organism. Although a select group of attributes that included the codon adaptation index, probability of expression in inclusion bodies, N-terminal disorder, and G + C content (filtered) were better at discriminating between positive and negative sets, algorithm performance was better when all 21 attributes (unfiltered) were used. Performance scores (sensitivity, specificity and area under the curve) from GenSET Phase 1 were better than those reported for six published methods. More importantly, GenSET Phase 1 ranked more known effectors (70.3%) in the top 40 ranked proteins and predicted 10–80% more effectors than three available programs in three of the four organisms tested. GenSET Phase 2 predicted 43.8% effectors in the top 40 ranked proteins when tested on four related or unrelated organisms. The lower prediction rates from GenSET Phase 2 may be due to the presence of different translocation signals in effectors from different T3SS families.

**Conclusions:**

The species-specific GenSET Phase 1 method offers an alternative approach to T3SS effector prediction that can be used with other published programs to improve effector predictions. Additionally, our approach can be applied to predict effectors of other secretion systems as long as these effectors have translocation signals embedded in their sequences.

**Electronic supplementary material:**

The online version of this article (doi:10.1186/s12864-016-3363-1) contains supplementary material, which is available to authorized users.

## Background

Many gram-negative bacteria possess contact-dependent type III secretion systems (T3SSs) that translocate diverse effector proteins from the bacterial cytosol into host cells [1, 2]. Effectors secreted by T3SSs play essential roles in the host-pathogen interactions by mimicking host proteins in structure and function, and by suppressing the host innate immunity [3]. Since effectors are crucial to the virulence of pathogenic bacteria, identifying and characterizing effectors is critical to our understanding and prevention of animal and plant diseases.

Improved sequencing technologies and the exponential growth in genomic and proteomic databases have opened up new and faster ways to identify and characterize effectors [4, 5]. Use of computational approaches to accurately predict novel effectors scattered in the genomes of T3SS-containing bacteria is an exciting area of research [4, 6]. Machine learning is an area of Artificial Intelligence in which computer programs are used to identify patterns and distinguish between different classes of objects resulting in phylogenetic tree construction, protein function prediction, and recognition of translocation signals in effectors [4, 7, 8].

Researchers have used homology sequence comparisons and machine learning algorithms to identify novel effectors in genomes from unannotated nucleotide and peptide sequences [4, 9]. However, designing a prediction program is difficult because effector features or attributes have not been well defined [4, 6]. One of the earliest effector prediction software programs, EffectiveT3, analyzed amino acid composition and secondary structure properties in the N-terminus of effectors and was used to predict effectors from several animal and plant pathogens [10]. Subsequent *in silico* approaches such as T3SEpre, SIEVE, and BPBAac have used machine learning to train algorithms on features such as G + C content, protein solvent accessibility, and position preference of the N-terminus amino acids [11–13]. These methods used T3SS effectors from unrelated genomes to generate the positive data sets. Although each of these methods identified putative effectors, the methods are not effectively and efficiently applicable across different bacterial species and are difficult to customize to a specific species.

In contrast to the other methods, Sato et al. [14] developed a meta-analytical approach to predict effectors from features derived from two genome sequences through machine learning. The resulting effectors were further enriched by secondary filters such as co-expression analysis. This program performed better than the BPBAac, SIEVE, and EffectiveT3 methods in predicting T3SS effectors in *Salmonella enterica* serovar Typhimurium (*S.* Typhimurium). Although Sato’s program had good predictive power, the program did not show significant improvements in accuracy. We propose that new and increased attribute combinations, together with a refinement of the positive set composition, can lead to the development of an even more accurate program. Additionally, it is necessary to develop a program that can be customized to different bacterial genomes for species-specific effector predictions.

We used a comprehensive list of 21 effector attributes (Table 1) to establish an effective machine learning method of predicting T3SS effectors called Genome Search for Effectors Tool (GenSET). In GenSET Phase 1, we used known effector and non-effector sequences from one bacterium to train five machine learning algorithms. A voting (trained) algorithm was then applied to predict effectors included in the testing set. This approach was species-specific and was successfully applied to the genomes of four different organisms. In GenSET Phase 2, we trained the five algorithms on a combined list of known effectors and non-effectors from two related bacteria with well-studied T3SS effectors, namely *S.* Typhimurium and *Shigella dysenteriae*. The voting algorithm was then used to predict effector sequences in the genomes of four different bacteria that were not used in the original training. The GenSET Phase 1 approach improves effector prediction for any species, is easy to apply, and can be used in well-studied as well as less-studied bacteria. The resulting candidates can then be tested further in wet bench experimental validation. The GenSET Phase 2 approach can be used to predict T3SS effectors in less studied organisms albeit with low prediction rates that may be explained by the presence of different families of T3SSs in different organisms.

**Table 1:** A summary of all 21 attributes used in the project

Program	Attribute	Full length or N_30_ region
Pepstats^a^	Peptide properties: tiny, small, aliphatic, aromatic, polar, non-polar, charged, basic, acidic Molecular weight	N_30_ region
	Charge A_280_ molar extinction coefficient A_280_ molar extinction coefficient (1 mg/ml) Probability of expression in inclusion bodies (PEPIB)	Full length N_30_ region N_30_ region N_30_ region N_30_ region
CAI^a^	Codon adaption index	Full length
ProtParam^b^	Isoelectric point Instability index Aliphatic index GRAVY Score	Full length N_30_ region N_30_ region N_30_ region
POODLE-S^c^	N-terminal disorder (N_30_ disorder)	N_30_ region
This study	G + C content	Full length

Web links of the above programs

^a^ http://emboss.sourceforge.net/

^b^ http://web.expasy.org/protparam/

^c^ www.cbrc.jp/cbrc-software

## Results

### Attribute selection

**Fig. 1:**
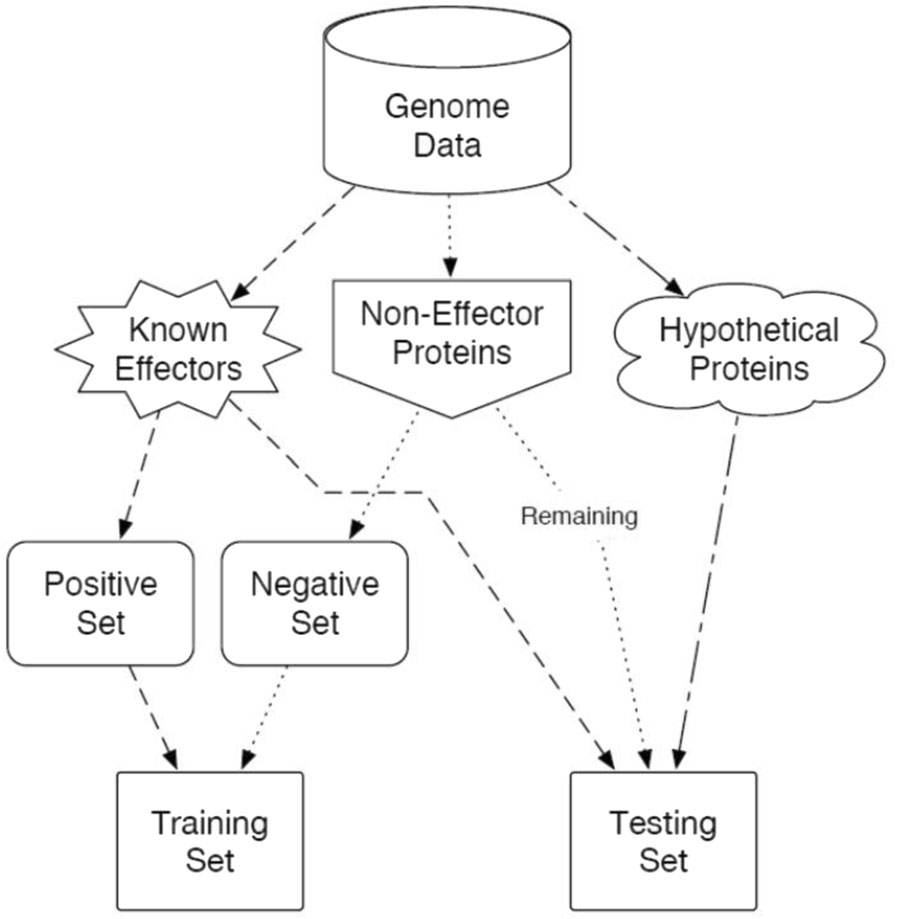
An Overview of GenSET Phase 1 selection of the training and testing sets for T3SS effector prediction. Protein or nucleotide sequences from each genome were grouped into three categories that included (i) all known T3SS effectors, (ii) non-effectors including non-T3SS annotated proteins, and (iii) all unannotated hypothetical proteins including all T3SS-related proteins. Fifteen randomly picked effectors (*E. coli*, *S. dysenteriae,* and *S.* Typhimurium) or 21 effectors (*P. syringae*) from (i) became the positive set. The negative training set was 10-fold larger group of non-effector randomly selected from (ii) of the same genome. GenSET was trained on the positive and negative sets (training set) using unfiltered attributes and filtered attributes and then applied to all remaining sequences of the whole genome (testing set)

GenSET Phase 1 generated data sets based on the genomes of the four test organisms (Fig. 1). All 21 attributes were used to set up a data set for machine learning called the unfiltered set (Table 1). At the same time, attributes that gave a good separation between the positive set and the negative set were used in the filtered set. In GenSET Phase 1, four attributes gave good separation between the positive and negative sets were selected for each organism, except for *Pseudomonas syringae* where eight attributes gave good separation and were selected using WEKA (Table 2; Additional file 1). The filtered set included at least three out of the four attributes (PEPIB, CAI, N_30_ disorder, and G + C content) for each organism. In GenSET Phase 2, eight attributes (aliphatic, acidic, PEPIB, instability index, molecular weight, CAI, G + C content, and N_30_ disorder) were selected for the filtered set. The means and standard deviations, for each attribute, were calculated and the values of the positive and negative sets compared. In general, the statistical analysis of the attributes correlated with the feature selection programs used in this study (Table 2).

**Table 2:** Statistical analysis of attributes chosen by the feature selection methods for the four organisms. Four attributes (PEPIB, CAI, N_30_ disorder, and G + C content) out of the 21 appeared in at least three of the four organisms tested. Actual values for all attributes are given in Additional file 1)

Organism	Attribute	Positive set		Negative set	
		Average	SD^b^	Average	SD
*E. coli*	Non-Polar	48.67	8.89	58.42	10.70
	PEPIB	0.86	0.07	0.77	0.14
	CAI	0.58	0.02	0.71	0.06
	N_30_ disorder	0.53	0.11	0.38	0.13
*P. syringae*	Tiny	41.11	9.45	30.32	9.09
	Charge^a^	0.93	1.25	0.60	2.40
	pI^a^	7.74	1.42	6.71	1.76
	PEPIB	0.90	0.11	0.74	0.16
	Aliphatic index	61.19	18.33	100.96	32.15
	CAI	0.51	0.06	0.68	0.07
	G + C content	51.62	5.77	59.04	3.91
	N_30_ disorder	0.67	0.07	0.41	0.14
*S. dysenteriae*	Non-Polar	45.11	9.07	56.09	10.11
	Tiny^a^	24.22	10.87	29.16	9.29
	G + C content	34.62	1.80	51.65	3.70
	N_30_ disorder	0.47	0.12	0.22	0.13
*S.* Typhimurium	PEPIB	0.88	0.12	0.74	0.21
	Instability index	65.21	22.55	38.43	22.54
	CAI	0.58	0.04	0.69	0.05
	G + C content	43.99	6.18	52.18	5.74

^a^Attributes that are not statistically different between the positive and negative sets

^b^ *SD* standard deviation

The five attributes that gave good discrimination between positive and negative sets in the four test organisms for both GenSET Phase 1 and 2 included the non-polar, PEPIB, CAI, N_30_ disorder and G + C content. All effector proteins, except those for S. dysenteriae, had N_30_ disorder average values above 0.50 for the positive and below 0.50 for the negative sets, respectively (Table 2, Additional file 1). Values above 0.50 represent disordered regions [15]. The positive set for *S. dysenteriae* had values below the disorder threshold, but it should be noted that the average values for the negative sets were also significantly lower than those calculated for other organisms. Although some workers have reported prediction of T3SS effectors based on the N-terminal disorder [15], this feature is rarely used in the literature. Attributes utilizing the nucleotide sequences, CAI and/or G + C content, gave positive and negative set values that were statistically different in all organisms. Similarly, two attributes that utilized protein sequences, non-polar and/or PEPIB values, also gave positive and negative set values that were statistically different in the four organisms (Table 2).

### Algorithm performance

Algorithm performance was evaluated by calculating the TPR, SPC, PPV, and AUC values (Table 3, Additional file 2). Generally, algorithm performance was slightly better with unfiltered rather than with filtered attributes. Therefore, the unfiltered attribute values were used in subsequent analyses for both Phase 1 and 2. AUC is a general bench mark for algorithm performance and our AUC scores for all algorithms were high in Phase 1 (above 0.900 for most organisms) except for SVM which gave AUC scores below 0.750 for *S.* Typhimurium (Additional file 2). We were most concerned with the sensitivity (TPR) of the methods since this measured whether or not the algorithms would be able to pick out effectors from the genomic haystack. Excellent TPR values (1.000) were recorded for three organisms in Phase 1 (*S.* Typhimurium, *S. dysenteriae*, and *Escherichia coli*) whereas a value of 0.750 was recorded for *P. syringae*. Specificity (SPC) values were also very good for all organisms tested in Phase 1 and ranged from 0.962 to 0.989; SPC measures the algorithms ability to correctly predict non-effector proteins. Precision values (PPV) on the other hand were low (ranging from 0.025 to 0.300). Further fine-tuning or optimization of the algorithm parameters can be done later to increase precision. In general, GenSET Phase 1 algorithms performed very well on all four organisms, as indicated by high TPR, SPC, and AUC scores. The bigger positive training set (21) in *P. syringae* had similar or lower performance values when compared to those in other organisms where smaller positive training sets (15) were used (Table 3).

**Table 3:** Average performance of the algorithms on the four organisms using unfiltered (U) and filtered (F) attributes. The PPV (positive predictive value or precision), TPR (True positive rate or sensitivity), SPC (specificity or true negative rate), and AUC (area under the curve) values were calculated using the trained (voting) algorithm (actual values for all algorithms are given in Additional file 2)

GenSET	Organism/Attributes^a^		PPV	TPR	SPC	AUC
Phase 1	*E. coli*	U	0.068	1.000	0.980	0.998
		F	0.082	1.000	0.984	0.993
	*P. syringae*	U	0.300	0.750	0.989	0.988
		F	0.520	0.542	0.997	0.970
	*S. dysenteriae*	U	0.086	1.000	0.984	0.999
		F	0.106	1.000	0.987	0.997
	*S.* Typhimurium	U	0.025	1.000	0.962	0.987
		F	0.022	1.000	0.956	0.984
Phase 2	*E. coli*	U	0.112	0.950	0.957	0.980
		F	0.132	0.800	0.970	0.979
	*Y. pestis*	U	0.429	0.273	0.999	0.943
		F	0.000	0.000	0.999	0.882
	*P. syringae*	U	0.330	0.800	0.982	0.981
		F	0.337	0.711	0.984	0.976
	*S. fredii*	U	0.364	0.444	0.998	0.967
		F	0.000	0.000	0.999	0.955

^a^The voting algorithm used unfiltered (U) attributes (utilize all 21 attributes) and filtered (F) attributes (utilizing a subset of attributes selected by the feature selection methods)

The Phase 2 scores of the four organisms for AUC (0.943–0.981) and SPC (0.957–0.999) were comparable to the values observed in Phase 1, but the scores for TPR (0.273–0.950) were lower than those from Phase 1 (Table 3). On the other hand, the scores for PPV (0.107–0.429) were mostly higher than those from Phase 1. PPV and TPR scores of zero were recorded for *Yersinia pestis* and *Sinorhizobium fredii* using the filtered attributes. These results further suggest that the voting algorithm performed better on unfiltered attributes than on filtered attributes. In general, the voting algorithm was able to give a good indication of the average performance of algorithms for the organisms in both Phase 1 and 2.

### GenSET Phase 1 performance and effector predictions

The ability of the algorithms to correctly predict known effectors from the testing set was evaluated using filtered and unfiltered attributes (Table 4 and Additional file 3). Using unfiltered attributes for the four bacteria, GenSET Phase 1 correctly identified 53.3*–*88.9% (average: 70.3%) of the known effectors in the top 40 positive prediction for each organism: *P. syringae* (53.3%), *S. dysenteriae* (55.6%), *E. coli* (83.3%), and *S.* Typhimurium (88.9%). The overall prediction averaged 78.6% for the unfiltered attributes (Table 4). The bigger positive training set in *P. syringae* had similar or lower effector prediction rates when compared to those in other organisms where smaller positive training sets were used (Table 4).

**Table 4:** GenSET prediction of known effectors that were included in the testing set. The number of known effector (and percentage) in the top 40 proteins candidates and the overall prediction by GenSET are shown (see Additional file 3 for all top 40 proteins predicted to be T3SS effectors)

GenSET	Organism	Unfiltered set		Filtered set	
		Top 40	Overall^c^	Top 40	Overall
Phase 1	*E. coli*	5/6^b^ (83.3%)	5/6 (83.3%)	4/6 (66.7%)	5/6 (83.3%)
	*P. syringae*	16/30 (53.3%)	16/30 (53.3%)	13/30 (43.3%)	13/30 (43.3%)
	*S. dysenteriae*	5/9 (55.6%)	8/9 (88.9%)	5/9 (55.6%)	5/9 (55.6%)
	*S.* Typhimurium (SPI-2)	8/9 (88.9%)	8/9 (88.9%)	6/9 (66.6%)	6/9 (66.6%)
Phase 1 Average^a^	All organisms	70.3%	78.6%	58.1%	62.2%
Phase 2	*E. coli*	14/21 (66.7%)	19/21 (90.5%)	12/21 (57.1%)	16/21 (76.2%)
	*Y. pestis*	2/8 (25%)	2/8 (25%)	0/8 (0%)	0/8 (0%)
	*P. syringae*	20/51 (39.2%)	36/51 (70.6%)	20/51 (39.2%)	32/51 (62.7%)
	*S. fredii*	4/9 (44.4%)	4/9 (44.4%)	0/9 (0%)	0/9 (0%)
Phase 2 Average	All organisms	43.8%	57.6%	24.1%	34.7%

^a^Average scores were calculated by averaging the unfiltered and filtered percent values of the four organisms

^b^Effector prediction rates were calculated by the number of effectors in the top 40 positive prediction over the total number of known effectors in the testing set

^c^Overall denotes all confirmed effectors proteins that were predicted to be effectors with true probabilities ≥ 0.5

### GenSET Phase 2 performance and effector predictions

During optimization of attribute selection, it was observed that *S. dysenteriae* and *S.* Typhimurium had only one overlap (G + C content) in their selected attributes (Table 2). However, the combined attributes from the two organisms (training) had a decent overlap in the selected attributes for the testing organisms such as *E. coli*, and *P syringae*. Thus, effectors of *S. dysenteriae* and *S.* Typhimurium were used for Phase 2 machine learning before applying the trained algorithms to four other organisms. This approach was successful in ranking 66.7 and 39.2% of effectors in the top 40 prediction in *E. coli,* and *P. syringae* respectively (Table 4). We extended the prediction to two other less-studied bacteria, namely *Y. pestis* and *S. fredii*, and ranked 25 and 44.4% respectively, in the top 40 positive prediction (Table 4 and Additional file 3) using unfiltered attributes. Overall, the prediction rate for GenSET Phase 2 averaged at 43.8% for the top 40 prediction for all four organisms and 57.6% for the overall prediction using unfiltered attributes. No effectors were ranked in the top 40 positive predictions for *Y. pestis* and *S. fredii* for the filtered attributes (Table 4 and Additional file 3). In general, GenSET Phase 1 had better top 40 prediction accuracy than Phase 2 for *E. coli* and *P. syringae* (Table 4).

### GenSET compared to other programs

The performance of GenSET Phase 1 and Phase 2 was compared to self-reported performances of six other existing effector identification programs (Table 5). The GenSET Phase 1 top 40 positive prediction values on the four organisms were also compared to those of the different programs on the same four organisms used in this study (Table 6). GenSET Phase 1 performed better in sensitivity (TPR), specificity (SPC), and area under the curve (AUC) values when compared to the six published programs (Table 5). GenSET Phase 2 performed better than GenSET Phase 1 with respect to SPC values but had slightly lower TPR and AUC values. More importantly, GenSET performed better than other programs in predicting many more T3SS effectors (Table 6). Among the six published programs listed in Table 5, only EffectiveT3, T3MM, and BPBAac were available or accessible. Protein sequences of the proteomes of *S. dysenteriae*, *E. coli*, *P. syringae*, and *S.* Typhimuirium were keyed into the above programs separately to obtain the top 40 positive prediction (Table 6 and Additional file 4). For the scoring of effector prediction, we only included “true effector” and did not count other T3SS proteins such as apparatus and translocon proteins as effectors. GenSET Phase 1 had higher effector prediction rates than the three established programs for *E. coli* (by 26% compared to the closest program), *P. syringae* (by 14%) and *S.* Typhimurium (by 59% for SPI-2 effectors). In the case of *S. dysenteriae*, our results were better than those from EffectiveT3 and BPBAac but were lower than those from T3MM (by 7%). Further, the GenSET method is T3SS family specific in that GenSET Phase 1 predicted more SPI-2 effectors than SPI-1 effectors when compared to the other methods (Table 6). In conclusion, our GenSET Phase 1 method offers an alternative approach for T3SS effector prediction and was proven to predict more effectors than some other established programs. We have included a step by step procedure for researchers to check the accuracy of the method for their target organisms (Additional file 5).

**Table 5:** GenSET performance was compare to six other machine learning programs using the average TPR (sensitivity), SPC (specificity), and AUC (area under the curve) values obtained with unfiltered attributes from the four organisms. GenSET 1 performed better than the other six programs in all areas whereas GenSET 2 gave better specificities

Program	TPR	SPC	AUC	Reference
EffectiveT3	~0.710	~0.850	0.85–0.86	[10]
T3MM	~0.839	~0.903	N/A^c^	[16]
SIEVE	0.9	0.88	0.95–0.96	[11]
BPBAac	~0.910	~0.974	0.989	[12]
T3SEpre	0.927	0.945	N/A	[13]
Meta-analytic	~0.90	~0.90	**0.993**	[14]
GenSET 1^a^	**0.938** ^b^	**0.979**	**0.993**	This study
GenSET 2	0.617	**0.984**	0.968	This study

^a^GenSET 1 and GenSET 2 denote average results from Phase 1 and Phase 2 respectively

^b^Bold number denotes the highest value in a given column

^c^ *N/A* Not applicable or not available

**Table 6:** GenSET 1 T3SS effector prediction on four organisms were compared to three other available machine learning programs. GenSET 1 performed better than other programs in three out of the four organisms tested except for *S. dysenteriae* (see Additional files 3 and 4 for the actual data)

	Top 40 positive prediction out of total confirmed effectors			
Program^a^	*S. dysenteriae*	*E. coli*	*P. syringae*	*S*. Typhimurium
EffectiveT3	7/24 (29.2%)	11/21 (52.4%)	9/51 (17.7%)	2/24 (8.3%)^d^ 1/8 (12.5%)^e^
T3MM	**15/24 (62.5%)** ^**c**^	5/21 (23.8%)	21/51 (41.2%)	4/24 (16.7%)^d^ 5/8 (62.5%)^e^
BPBAac	13/24 (54.2%)	12/21 (57.1%)	20/51 (39.2%)	7/24 (29.2%)^d^ 6/8 (75.0%)^e^
GenSET 1^b^	5/9 (55.6%)	**5/6 (83.3%)**	**16/30 (53.3%)**	**8/9 (88.9%)** ^d^ 4/8 (50%)^e^

^a^Other programs namely SIEVE, T3SEpre, and Meta-analytic were not available or accessible at the time of investigation

^b^For the GenSET 1 method, 15 or 21 effectors were taken out from the total effectors as the positive data sets. Thus, the totals were less in numbers when compared to others

^c^Bold number denotes the highest value in a given column

^d^Top 40 positive prediction for SPI-2 effectors

^e^Top 40 positive prediction for SPI-1 effectors

## Discussion

This study served as a proof of concept for the GenSET method for accurate T3SS effector prediction. We used a wide range of effector attributes to build predictive models through machine learning that could identify differences between effector and non-effector data sets. In GenSET Phase 1, our approach significantly increased effector prediction accuracy for the majority of species tested (3 out of 4) (Tables 4 and 6). The method predicted 10 to 80% more effectors in the top 40 proteins than the other established methods in three out of four test organisms (Table 6). The method was customized to four specific organisms and can be applied to other organisms to predict effectors in individual genomes. The GenSET method can therefore reduce the number of labor-intensive wet-lab validation experiments for effector prediction.

In GenSET Phase 1, we used 15 effectors for *E. coli*, *S. dysenteriae*, and *S.* Typhimurium or 21 effectors for *P. syringae* for the machine learning; whereas, in GenSET Phase 2, we used 30 effectors (15 each) from two related organisms in the family of Enterobacteriaceae. A bigger positive training set did not improve the performance values (Table 3) or the prediction rate (Table 4) when compared smaller positive training sets. Thus, the minimum number for the positive set could be set at 15 effectors. The strength of our GenSET method is in the use of a smaller positive sets such as 15 confirmed effectors for Phase 1 and 30 for Phase 2, and the potential to customize the method for species-specific prediction in any genome. In contrast, other published programs such as BPBAac [12], T3MM [16] and EffectiveT3 [10], used a pool of heterogeneous effectors from many genomes to construct their positive sets. These genomes were from phylogenetically diverse organisms with different T3SSs families that prevented the customization of the pooled effector data set for species-specific predictions.

Seven different families of T3SSs in gram-negative bacteria have been proposed based on the phylogram of ATPases. The families include SPI-1 and SPI-2 in *S.* Typhimurium, Ysc in *Y. pestis*, Hrp 1 in *P. syringae*, Hrp 2 in *Xanthomonas campestris*, chlamydiales and rhizobiales [17, 18]. These T3SS families may have different translocation signals embedded in their respective effectors. One problem arising from combining effectors from different families of T3SSs to create a generic classifier for machine learning is that different effectors may emphasize different attributes and this may introduce a strong bias into the classifier and reduce the performance of generic effector prediction algorithms when applied to specific organisms. For example. Sato et al. [14] defined the training set with effectors from single genomes of two organisms, *S.* Typhimurium and *P. syringae*. One disadvantage to this approach was that the SVM machine learning was performed on one organism and then applied to another unrelated organism for effector prediction. Similarly, Yang et al. [19] based their classifier training on *P. syringae* and then applied the method to rhizobial strains. Different effectors from different families of T3SSs may have different translocation signals. Therefore, combining effectors from unrelated organisms in machine learning may reduce prediction rates. The species-specific approach used in GenSET Phase 1 eliminates these biases and improves T3SS effector prediction in individual species. Indeed, our GenSET Phase 1 had better prediction rates that ranked an average of 70.3% of effectors in the top 40 positive prediction and 78.6% of the effectors overall (Table 4). These prediction rates were 10 to 80% better than other established methods on three out of the four organisms tested (Table 6). Although Yang et al. [19] pooled effectors from three different strains of the same species of *P. syringae* to make the positive set homogenous, the trained algorism was applied to unrelated species or families of T3SSs. Indeed, a trained algorithm called TREEE based on machine learning on *P. syringae* performed poorly when applied to *S.* Typhimurium [20].

Additionally, the mixing of two different types of T3SS effectors in the training set, such as SPI-1 and SPI-2 of *S.* Typhimurium, may have reduced the performance of the SVM training by Sato et al. [14]. In the GenSET Phase 1 approach, our machine learning was not only species- specific but also T3SS family-specific in order to increase prediction accuracy. For example, our machine learning on *S.* Typhimurium was specific to SPI-2 family effectors and predicted 88.9% of known effectors in the top 40 positive prediction (Table 4). GenSET was thus successful in predicting effectors in a species-specific manner as long as the organism had a minimum size of known effector population, such as 15 effectors. Our results strongly suggest that GenSET Phase 1 can be customized to any organisms and we have successfully applied it to four organisms in this study.

In order to investigate the universal application of GenSET to less-studied organisms with fewer identified effectors, we combined positive sets from two closely related organisms (*S.* Typhimurium and *S. dysenteriae*) in the GenSET Phase 2 and then applied the algorithm to *E. coli*, *P. syringae*, *Y. pestis* and *S. fredii*. It should be noted that *E. coli* belongs to the same T3SS family as the *Salmonella* species used to construct the training set whereas *P. syringae*, *Y. pestis* and *S. fredii* belong to different families of T3SSs [17, 18]. This may explain why the top 40 positive prediction rates and sensitivity values for the other three organisms were lower than those observed for *E. coli* (Tables 3 and 4). The lower sensitivity values suggest slightly different translocation signals in the training and testing data sets. However, these prediction rates are significant for an initial screening tool to reduce the down time spent in wet bench experiments for T3SS effector identification.

The GenSET Phase 1 approach had the highest prediction accuracy, was T3SS family-specific, and has potential to be universally applicable to any organisms. Some of the effectors of *S.* Typhimurium can be translocated using both SPI-1 and SPI-2 apparatuses [21]. Therefore, it was not surprising to see SPI-1 effectors identified using a SPI-2 specific machine learning in *S.* Typhimurium in GenSET Phase 1. GenSET Phase 1 method predicted eight out of nine (89%) SPI-2 effectors in the top 40 positive prediction (Table 4). We also picked four out of eight (50%) SPI-1 effectors in the top 40 positive prediction (Additional files 3 and 4). Furthermore, our method not only predicted effectors in less-studied organisms but was able to predict novel effectors in well-studied organisms. For example, in the top 40 ranked effector prediction from *S.* Typhimurium strain LT2 by GenSET, we were able to pick out about 30 hypothetical proteins. Some of these hypothetical proteins may be novel effectors that await further characterization.

GenSET used five different algorithms and a voting algorithm for machine learning on organisms with different effector population sizes and compositions of positive sets. The use of the voting algorithm is advantageous in that this can increase error tolerance. If one algorithm is completely off target in its predictions and the other four worked well, the averaging process reduces the impact of the poorly performing algorithms. For example, the filtered SVM algorithm on *S.* Typhmurium did not pick out any of the known effectors but because the other algorithms picked out the effectors, the voting algorithm ended up predicting them to be effectors (Additional file 3). In comparison, other programs only used one to three algorithms for the training. For example, SVM was used for the meta-analytical approach [14], SIEVE [11] and BPBAac [12]. SVM, generalized linear model and RandomForest were used for the T3MM by Wang et al. [16].

We started with 21 attributes (features) and employed attribute selection methods to define a subset of attributes called the filtered sets. Other published methods (i.e. SIEVE and T3SEpre) concentrate on a few attributes, such as G + C content and amino acid composition [11, 13]. However, we prefer to use a comprehensive list of attributes so that we can cover all the possible characteristics. We looked at peptide property to understand their physico-chemical nature; this property has been well examined by other researchers [9–11]. We also examined molecular weight, charge and pI as effectors are generally small in size and have a charged residue bias [4]. Other features used were related to the structures and environments, and included stability of the protein using aliphatic index and N-terminal disorder, solubility measure (PEPIB), hydropathy values (GRAVY score), and G + C gene content bias.

In general, the unfiltered sets performed better than the filtered sets in all organisms. This feature possibly works well for our species- and T3SS family-specific approach and can be adaptable to other organisms. Possible future directions to further improve this project will include researching and evaluating additional attributes that can be used in this method. The goal is to develop an exhaustive list of attributes that can characterize translocation signals embedded in the effector sequences. Additionally, we can fine-tune the parameters of the machine learning algorithms in order to increase the precision and reduce the number of false positive predictions. For example, we can increase the length of N-terminal sequence from N_30_ to N_50_ or longer as suggested by Wang et al. [13], or we can increase the size of the training sets.

It is not clear to us why the unfiltered attribute sets performed better than the filtered sets in general. One possible explanation is that perhaps there are clusters of effectors that are more related to each other than they are to other effectors. Indeed effectors could be classified and grouped under some common families [3]. If the majority of the effectors are inside such a relation cluster, this would create a bias in the feature selection algorithms in selecting attributes that pinpoint that relation cluster. The use of the unfiltered set can cover more such clusters and thus compensate for effectors that lie outside that relationship clusters. Perhaps there are several such relational clusters of effectors within the same or in different organisms especially since about 30% of each genome is still unannotated.

## Conclusions

The GenSET Phase 1 approach is user-friendly, performs efficiently and effectively when compared to other established programs, and can be applied to any bacterium. Third-party programs were used to produce data sets and attribute analysis to allow biologists with little computing background to predict effectors in bacteria genomes. We have included a step by step approach for setting up GenSET as supplementary information (Additional file 5). This information can be customized for other gram-negative organisms. With further refinements, a software framework can be developed that would turn the GenSET method into a single stand-alone application for researchers to use. Future work on GenSET Phase 2 will seek to improve the effector prediction rate for the less studied organisms. Researchers need to take into account the T3SS family to which the organisms of interest belongs when developing prediction algorithms. Perhaps we can setup seven different trained algorithms for seven different families respectively to increase the low prediction rate. Furthermore, the GenSET approach can extended to predict other effectors in other secretion systems such as type IV (T4SS) and type VI (T6SS) as long as there are specific signals embedded in the effectors for machine learning.

## Methods

### Selected bacterial species and their effectors

The six gram-negative bacterial genomes chosen in this study have a large number of well characterized T3SS effectors that made them ideal candidates for this bioinformatics project [3, 5, 19–24]. The selected bacteria have different habitats and included three food- or water-borne pathogens belonging to the family of Enterobacteriaceae, one plant pathogens, one plant symbiont, and one vector-borne animal pathogen. These bacterial strains rely on T3SSs to infect host cells [19–24]. Characterized effector sequences from these bacteria were retrieved from NCBI databases (http://www.ncbi.nlm.nih.gov/). The lists of “true” effectors of these genomes did not include known extracellular apparatus proteins such as translocons and needle proteins for machine learning (see Additional file 6 for the lists the effectors used in this study). These true effector lists were also compared to recent literature to confirm their identities [19–24].

*E. coli* O127:H6 strain E2348/69 is a food-borne pathogen that infects intestinal walls in humans and causes watery diarrhoea, vomiting, and fever. The 21 known T3SS effectors in this organism [23] were used (15 effectors in the positive training set and six in the testing set, Additional file 6). *S. dysenteriae* strain Sd197 spreads through contaminated food and water and targets the small intestines where it causes severe dysentery and colitis. The 24 known T3SS effectors in this organism [22] were used (15 in positive training set and 9 in testing set, Additional file 6). *S.* Typhimurium strain LT2 spreads through contaminated food and is the main cause of gastroenteritis in humans and a typhoid-like disease in mice. The bacterium has two different T3SSs, namely SPI-1 and SPI-2. Our study focused on the SPI-2 system because it shares similarities with the T3SSs in *E. coli*. The 24 known T3SS effectors (15 in positive training set and 9 in testing set, Additional file 6) that use the SPI-2 apparatus for translocation were used; eight other effectors use the SPI-1 apparatus for translocation [21]. *P. syringae pv. tomato* strain DC3000 infects tomato plants. It forms bacterial specks on infected plants and can lead to significant economic losses during tomato production. The 51 known T3SS effectors in this organism [20] were used (21 in positive training set and 30 in testing set, Additional file 6). *Y. pestis* strain CO92 is the causative agent for the bubonic plague. The eight identified T3SS effectors in *Y. pestis* [24] were used in the testing set (Additional file 6). *S. fredii* strain NGR234 is a plant symbiont that supplies nitrogen in many plant species. *S. fredii* uses T3SS for its symbiotic relationship and the nine known effectors from the organism [19] were used in the testing set (Additional file 6).

In this study, we took two approached to predicting T3SS effectors in the genomes of organisms. In GenSET Phase 1, our approach focused on developing an algorithm that was species-specific and was applicable to organisms with a large number of known T3SS effectors. The GenSET Phase 2 approach focused on developing an algorithm that could be used to predict effectors in less studied organisms, with a small number of known effectors.

### Overview of Phase 1 GenSET

Protein or nucleic acid sequences were divided into three categories: (I) all confirmed T3SS effectors, (II) all non-effectors, including all non-T3SS related annotated proteins, and (III) all unannotated hypothetical proteins or nucleic acids (including all T3SS related proteins) (Fig. 1).

The positive and negative sets were randomly generated from the above three categories by a pseudo-random process using the random.org web [25]. The positive set was generated by randomly selecting 15 or 21 effectors of the known effectors sequences from category (I). The negative set was randomly generated by selecting a 10-fold larger group than the positive set from the non-effector and non-T3SS related annotated sequences from category (II). The positive and negative sets were called the training set. The testing set contained all sequences not included in training set from categories (I) and (II), together with all category (III) sequences (unannotated hypothetical and T3SS related sequences). The training set was used to train the machine learning algorithms in GenSET. Five machine learning algorithms were used on the two data sets from each organism. A trained algorithm (voting) was then applied to the testing set to identify effectors (known and unknown) in the testing set. We included known effectors in the testing set to validate and to evaluate the performance of the algorithms.

The Waikato Environment for Knowledge Analysis (WEKA) machine learning program was used to analyze the data [26]. WEKA is a user friendly graphical workbench for machine learning that is targeted towards scientific research. We used the feature selection method in an attempt to generate more accurate attribute subsets for classification (filtered set). Three main categories of feature selection methods are available, the filter, wrapper, and embedded methods [27, 28]. The filter and wrapper methods were employed in this study. All 21 attributes were used as “unfiltered” set to measure algorithm performance (Table 1). Feature selection methods were then used to pick a subset of attributes as the “filtered” set to measure algorithm performance.

### Overview of Phase 2 GenSET

**Fig. 2:**
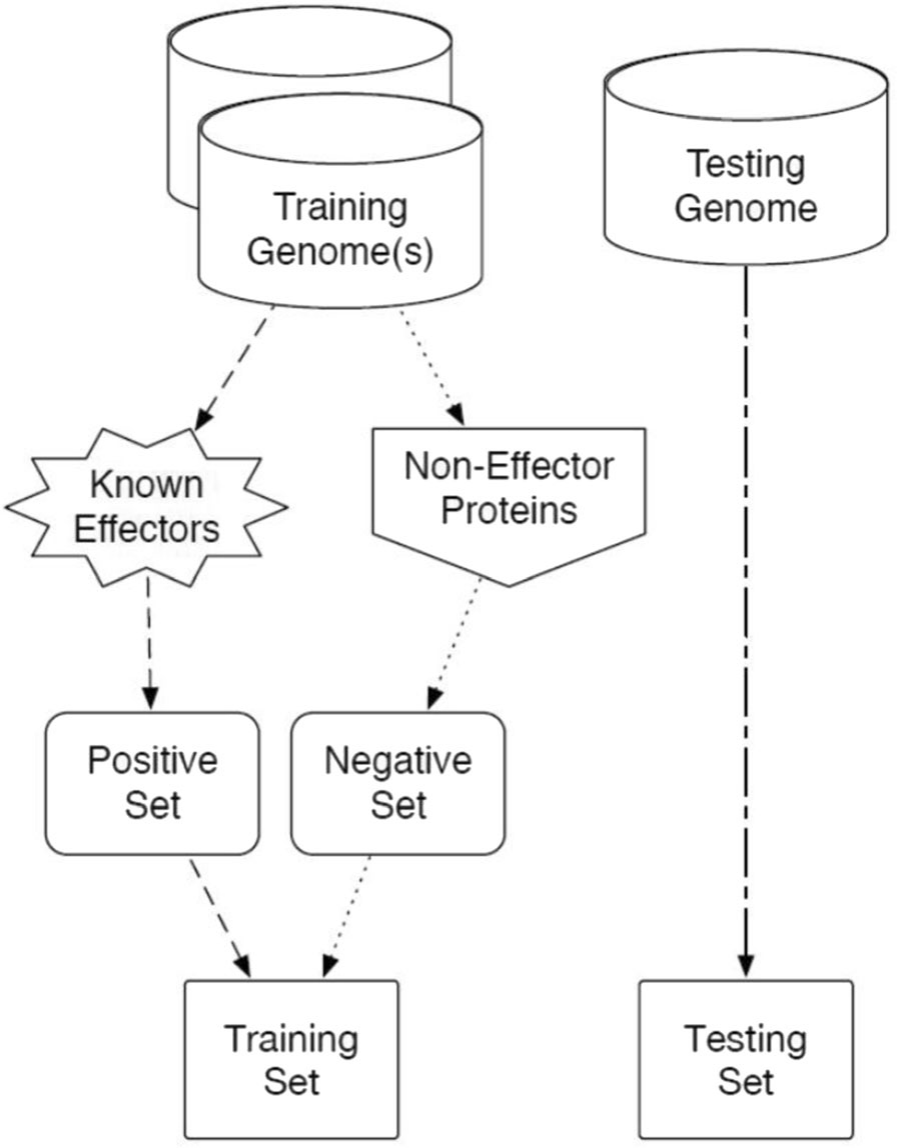
An Overview of GenSET Phase 2 selection of the training and testing sets for T3SS effector prediction. The positive set comprised 30 known effectors (15 each from *S. dysenteriae* and *S.* Typhimurium). The negative set was a 10-fold larger group of randomly selected non-effectors from the two organisms. Machine learning was similar to that in GenSET Phase 1 and used unfiltered and filtered attributes. The trained algorithm was applied to the genome of a third organism for T3SS effector prediction. The testing sets comprised all sequences in the test organism’s genome (i.e. *E. coli*, *Y. pestis*, *P. syringae*, and *S. fredii*)

Phase 2 GenSET was similar to Phase 1 except we combined effectors from two related organisms, *S.* Typhmiurium and *S. dysenteriae*, for machine learning (Fig. 2) and then applied the trained algorithm to other organisms not used in the initial training. The positive set contained 30 known effectors randomly selected from the two organisms (15 effectors each). The negative set was a 10-fold larger than the positive set of randomly selected sequences from the non-effectors sequences of the two organisms. The testing set included all sequences in the testing organism’s genome, namely *E. coli*, *S. fredii*, *Y. pestis*, or *P. syringae*. The same 21 attributes used in Phase 1 were also used in Phase 2 and the data reorganized to reflect the new training and testing sets. Algorithm performances on the “filtered” and “unfiltered” sets were also determined.

### Protein and nucleotide attribute gathering

We used third-party applications to generate the genomic and proteomic features (or attributes) data required for machine learning and classifications (Table 1). Custom Perl and Bash scripts were created to automate the submission and execution of these programs and to parse the returned results. The BioPerl library was used in many Perl scripts to handle the sequence operations and manipulations [29]. We also created a program to calculate the G + C content of nucleotide sequences.

The EMBOSS-Pepstats tool by the European Molecular Biology Open Software Suite (http://emboss.sourceforge.net/) was used to generate protein features. These included: (i) peptide properties (molar-percent composition of tiny, small, aliphatic, aromatic, polar, non-polar, charged, basic, and acidic amino acids), (ii) molecular weight, (iii) protein net charge, (iv) molar extinction coefficient at 280 nm, and (v) the probability of protein expression in inclusion bodies (PEPIB) rather than cytosol. The EMBOSS-CAI tool was used to generate the codon adaptation index (CAI). The ProtParam tool from ExPASy (Swiss Institute of Bioinformatics, http://web.expasy.org/protparam/) was used to generate the physical and chemical features: (i) isoelectric point, (ii) instability index, (iii) aliphatic index, and (iv) grand average of hydropathicity (GRAVY) score. POODLE-S tool developed by the Computational Biology Research Center in Japan (www.cbrc.jp/cbrc-software) was used to calculate the disorder values of the given protein sequence.

### Algorithms used in this study

Five algorithms were used for the machine learning using WEKA [26, 30]; for general information about the following algorithms refer to Larranaga et al. [8] and Witten and Frank [28]. (i) Bayesian Network, a probabilistic method directed at acyclic graph models based on random variables and their conditional dependencies. This algorithm is focused on attempting to minimize the cost of misclassification; (ii) Naive Bayes, a probabilistic model based on random variables that assumes independence between the variables and attempts to minimize the cost of misclassification; (iii) Logistic Regression, a statistical model that measures the correlation between a dependent classifier and one or more independent variables or attributes; (iv) Multilayer Perceptron (MLP), a form of artificial neural network algorithm that is good for data sets that are difficult to linearly discriminate between two classes; (v) Support Vector Machine (SVM), works by identifying patterns in the classification groups in its vector space and using these patterns to predict the classifications of unknown points. The sixth algorism was a trained (or voting) algorithm that averaged the probability given by the previous five algorithms for classifications.

### Algorithm validation and performance

Two types of algorithm validation methods were used in this study. The first was the 10-fold validation method used during the algorithm training stage. In this method, the training sample is divided into ten equal parts. One part is reserved while the other nine are used to train the algorithm. This process is repeated ten times, until each of the ten parts is used for validation. The results from the ten iterations are then combined into a single final model. The second validation method was the holdout method and was used during the testing stage [31]. In this method a sub-set of known effectors is left out of the training stage and is combined with the testing set. This gives performance feedback on how well the algorithm is able to identify known positive instances that were not involved in its training. Algorithm performance was based on calculated sensitivity (true positive rate; TPR), specificity (true negative rate; SPC), precision (positive predictive value; PPV), and area under the curve (AUC).

## Additional files

Additional file 1: Statistical analysis of the 21 attributes for the four organisms used in this studies. SD denotes standard deviation. Attribute values that have statistically significant differences between the positive and negative sets are highlighted. (XLSX 25 kb)
Additional file 2: Algorithm performance of five organisms using the filtered and unfiltered data sets for GenSET Phase 1 and Phase 2 studies. (PPV: precision value; TPR: sensitivity; SPC: specificity; AUC: area under the curve). (XLSX 1150 kb)
Additional file 3: Top 40 predictions of T3SS effectors for Phase 1 and Phase 2 by GenSET. Each protein in the testing sets were given a value (true probability) based on the training (voting) algorithm that takes the average of the probabilities given by the five algorithms. The proteins were then ranked based on size of the averaged true probability. Only the top 40 proteins with scores of 0.5 and above are listed. (XLSX 15 kb)
Additional file 4: Comparison of top 40 predictions of T3SS effectors in *S.* Typhimurium, *E. coli*, *P. syringae*, and *S. dysenteriae* by EffectiveT3, T3MM, BPBAac, and GenSET 1. EffectiveT3 program was from Arnold et al. 2009 [10]; T3MM program was from Wang et al., 2013 [16]; and BPBAac program was from Wang et al., 2011 [12]; GenSET data was from this study. (XLSX 47 kb)
Additional file 5: A brief protocol on the setting up GenSET for T3SS effector prediction customized for other gram-negative genomes. (DOCX 40 kb)
Additional file 6: Positive (effectors), negative (non-effectors), and testing sets of the five organisms that we used in the GenSET studies. (ALL Tags denotes all proteins encoded in the genome; Positive Set denotes known effector proteins used for training; Negative Set denotes non-effector proteins used for training; Testing Set denotes all proteins in the bacteria minus the Positive and Negative Sets; Positives in Testing Set denotes known effector proteins included in the Testing Set). (XLSX 48 kb)

## Abbreviations

AUC: Area under the curve
CAI: Codon adaptation index
GenSET: Genome Search for Effectors Tool
GRAVY: Score for hydropathy value
MLP: Multilayer perceptron
N_30_: First 30 amino acid at the N-terminal
PEPIB: Probability of expression in inclusion bodies
PPV: Positive predictive value or precision
*S.* Typhimurium: *Salmonella enterica* serovar Typhimurium
SD: Standard deviation
SPC: True negative rate or specificity
SVM: Support vector machine
TPR: True positive rate or sensitivity
Voting (trained) algorithm: Average probability given by 5 algorithms, namely Naïve Bayes, Logistic Regression, Bayesian Network, Multilayer Perceptron, and Support Vector Machine
