# Supplementary material for: Computational approach to predict species-specific type III secretion system (T3SS) effectors using single and multiple genomes
Source: BMC Genomics. 2016 Dec 19;17:1048. doi: 10.1186/s12864-016-3363-1 (PMC5168842; doi:10.1186/s12864-016-3363-1)
Supplement: Additional file 5: — A brief protocol on the setting up GenSET for T3SS effector prediction customized for other gram-negative genomes. (DOCX 40 kb) [file 12864_2016_3363_MOESM5_ESM.docx]

# Data Preparation

1. Download the chromosome and plasmid sequence libraries (both nucleotide and protein) for your desired organisms from NCBI (https://www.ncbi.nlm.nih.gov/home/tools.shtml). Click on the drop down menu, choose either ‘Nucleotide’ or ‘Protein’ sequences, then click “Create File”. Next, run the NCBI developed Perl script called “split_multifasta.pl” on each library. This will split the single multifasta files into individual files containing one nucleotide/protein sequence per file.
   - Ex: *perl split_multifasta.pl --input_file=seqs.txt*  *--output_dir=SplitSeqs --seqs_per_file=1*
2. Rename the files to <LocusTag>.fasta
   - Ex: YPO1111.fasta, YPO1112.fasta, YPO1113.fasta, etc…

# Building Data Sets

1. Compile a list of all confirmed effectors for your desired organism.
2. Randomize the order of the list. RANDOM.org is recommended: https://www.random.org/lists/?mode=advanced.
3. Remove at least 25% of the confirmed effectors from the randomized list for the testing set. The remaining 75% of the effectors will go into the positive training set.
4. Compile a list of all annotated (non-hypothetical) proteins that are not effectors.
5. Randomize this list using the above process.
6. The negative set was a 10-fold larger group than the positive set randomly selected from non-effectors.
   - Ex: If you have 15 known effectors in your positive set, you will select 150 proteins from this second randomized list.
7. All proteins that do not make up the positive and negative sets go into the testing set together with the 25% of confirmed effectors for validation and performance gauging purposes.

# Attribute Gathering

1. Once you have completed the above sets and broken down the sequence files into their respective sets (for both proteins and nucleotides), you will need to create a second truncated set. Using either a custom BioPerl script or tools provided with EMBOSS, you will need to create a second set of the protein sequences cropped to the first 30 amino acids on the N-terminal region. We require the two different protein sets because some attributes use the whole sequence while others use only the cropped sequence. Nucleotide sequences do not need a cropped set because the two attributes using nucleotides use the full-length sequence.
2. Next, install and configure the third part programs used in this study: a) EMBOSS Suite (http://emboss.sourceforge.net/), b) ProtParam (http://web.expasy.org/protparam/), and c) Poodle-S (<http://mbs.cbrc.jp/poodle/poodle-s.html>). ProtParam and Poodle-S are submitted through a web portal. However, it is **highly** recommended that you contact the Poodle-S developers for a copy of the software to install locally if you are going to analyze multiple organisms. See the respective websites for details on how to install and configure these programs.
3. ProtParam is the simplest program to start run. Use a simple Perl script to submit protein sequences to ProtParam and to record the response. This web service extremely robust and stable and could handle multiple sequences submitted within a short time.
4. Once installed on the local server, EMBOSS and potentially Poodle-S can be used for the attribute gathering process.
5. Two programs from EMBOSS were used; PepStats was applied to the full length and cropped protein sequences. CAI was applied to the full-length nucleotides but you must first use the CUSP program to build a Codon Usage Table. Both PepStats and CAI can only handle one sequence per execution but this can easily be automated using a small bash script.
6. Poodle-S comes with easy to follow instructions. All you need to do is run Poodle-S on the cropped sequences and parse the results files.
7. To calculate the G+C content of the nucleotides, we used a custom Perl script but you can easily find third party tools that can calculate the the G+C content.

# Machine Learning

For specifics on how to use Weka machine learning programs, please consult the user information located at: <http://www.cs.waikato.ac.nz/ml/weka/documentation.html>

1. Open up the Weka program and select the Explorer application.
2. The default tab on starting Weka Explorer is the “Preporecess” tab. On that tab page press the “Open file…” button and select the file that contains your training data. If you are using a filtered set of attributes, you can also remove the unused attributes on this tab.
3. Next move over to the “Classify” tab and choose the classifier that you want to use. We used the following classifiers in our research:
   1. Bayes/BaysNet
   2. Bayes/NaiveBayes
   3. Functions/Logistic
   4. Functions/MultilayerPerceptron
   5. Functions/SMO
   6. Meta/Vote
4. After choosing your classifier, select the “Use training set” radio button and press start. This will train the model and give you performance statistics on how the model did when being verified. We recommend that you save the trained model to ensure consistency in future runs and for archival purposes. Right clicking on the trained model that you want to save in the “Results List” pane and selecting the “Save Model” option.
5. To run a trained model, select the “Supplied test set” radio button and press the “Set…” button. This button will open up a new window for you to select the testing set data file. Now open the “More options…” window and ensure that “Output predictions” is selected for the prediction results of each protein in the testing set. Once completed, right click on the trained model and select “Re-evaluate model on current test set” and it will compare the testing set to the model and print out the predictions and related performance statistics.
